# Insight into the optoelectronic properties of designed solar cells efficient tetrahydroquinoline dye-sensitizers on TiO_2_(101) surface: first principles approach

**DOI:** 10.1038/s41598-018-29368-9

**Published:** 2018-07-20

**Authors:** Juganta K. Roy, Supratik Kar, Jerzy Leszczynski

**Affiliations:** Interdisciplinary Center for Nanotoxicity, Department of Chemistry, Physics and Atmospheric Sciences, Jackson State University, Jackson, MS 39217 USA

## Abstract

Seven ‘lead’ dye-sensitizers from Tetrahydroquinoline (THQ) family were proposed and designed based on the structural attributes *via* quantitative-structure property relationship (QSPR) modeling. They were screened rationally through different computational approaches to explore their potential applications as photosensitizers for dye-sensitized solar cells (DSSCs). Compelling photophysical properties such as electron injection driving force, electron injection time, and dye regeneration were studied for the isolated dyes under the DFT and TD-DFT frameworks. Index of spatial extent (S, D, and ∆q), the strength of charge transfer and separation along with the charge transfer process is explored. First principle approach including van der Waals density functional calculation of dye*@*TiO_2_ interface indicates that all of the designed dyes have optimal interfacial behavior. Bader charge analysis, partial density of state (PDOS), charge density and electrostatic potential difference calculation confirms that THQ7 and THQ9 are the most efficient dye-sensitizers. The other five designed dyes also possess the required properties to emerge as effective dye-sensitizers potentially better than those already utilized.

## Introduction

Captivating advantages of high molar extinction coefficient, flexible assembling, low-cost fabrication, and environmental cleanliness has helped metal-free organic dye-sensitized solar cells (DSSCs) to gain considerable interest as an alternative renewable energy source over the last decade^1–7^. In DSSC system, an organic photosensitizer is anchored to the wide band gap semiconductor and possesses a donor-π-acceptor (D-π-A) structure motif with well-developed intramolecular charge transfer (ICT) characteristics. The photoactive sensitizer of DSSCs absorbs sunlight in the form of the photon and photo excited dye injects electrons to the conduction band (CB) of the semiconductor such as TiO_2_. The performance of DSSCs relies on fundamental quantum properties like the lowest unoccupied molecular orbital (LUMO), the highest occupied molecular orbital (HOMO), and their distributions in the photosensitizers. Most significantly, the kinetics of the electron-transfer processes at the interface between the dye and the semiconductor as well the dye bound to the semiconductor surface and the hole-transporting substance^8,9^. Thus, it is imperative that the microscopic interfacial properties of organic metal-free dyes on TiO_2_ photoanodes need to be thoroughly investigated. This will elucidate the details of charge transfer and anchoring state at the interface, band gap offset of dyes, HOMO and LUMO shift, and their redistribution.

Computational approaches have become an integral part of research in material science following their successes in drug design and discovery process. *In silico* modeling to design, followed by quantum and electrochemical parameters calculation, represents the complete scheme for the rational solar cell development prior to experiment^10–13^. Our research group has efficiently employed quantitative structure-property relationship (QSPR) approach for modeling and virtual screening of polymer-based solar cells for the very first time^14^. It was followed by first QSPR modeling of arylamine organic dyes (AOD) for DSSCs explicit to cobalt electrolytes that were recently reported^5^. In our previous study, we have modeled 273 AODs from 11 diverse chemical classes explicit to iodine electrolyte followed by quantum chemical analysis employed to understand the primary electron transfer mechanism and photophysical properties. Further, identified features from QSPR models were applied in designing of 10 dyes - each for Tetrahydroquinoline (THQ), N,N-dialkylaniline and Indoline, respectively maintaining required electrochemical parameters with encouraging enhancement in predicted percent power conversion efficiency (PCE) value^13^. It is important to mention that finally, based on requisite electrochemical and photo-physical parameters seven lead dyes have been screened from the ten designed dyes for each chemical class. Expanding the research work to the next level, in the present study, we have investigated the seven THQ lead dyes to explore the interfacial properties as well as further photophysical properties of the isolated dyes. Compare to Rubidium based organic dyes; metal free organic dyes are receiving more endorsement to devise DSSCs due to the advantages of molecular engineering including the possibility of tuning donor-acceptor of dyes, their broad optical absorption, and exceptional electrochemical properties^15–17^.

Though a substantial number of studies have been directed towards metal-based organic dyes, only a limited work^6,18,19^ reported on non-metal organic dyes-sensitizers. Computational approaches focused on the semi-classical^20^ and Density Functional Theory (DFT) approaches combined with Time-Dependent DFT (TD-DFT)^12,21–24^, have been used to explore the electron injection process at the interface of metal and non-metal based organic dyes. First principle approaches were carried out to study the characteristics of the designed THQ dyes before and after the adsorption on TiO_2_ surface to compare qualitatively different aspects of the complex phenomena. Initially, we have analyzed the crucial parameters of the isolated dye like open-circuit voltage, short-current density, electron injection efficiency, reorganization energy, injection time, and intramolecular charge transfer (ICT) parameters by DFT and TD-DFT calculations. On the next level, structural and electronic properties, charge density and electrostatic potential energy differences of the adsorbed system, variations in the band gap and projected density of state were computed with the use of plane-wave DFT including Hubbard correction. The encouraging outcomes of the performed study allow selecting new efficient THQ dye sensitizers for DSSCs to obtain higher photo-to-electron conversion efficiency.

## Results

### Optoelectronic properties of isolated dyes

**Figure 1:**
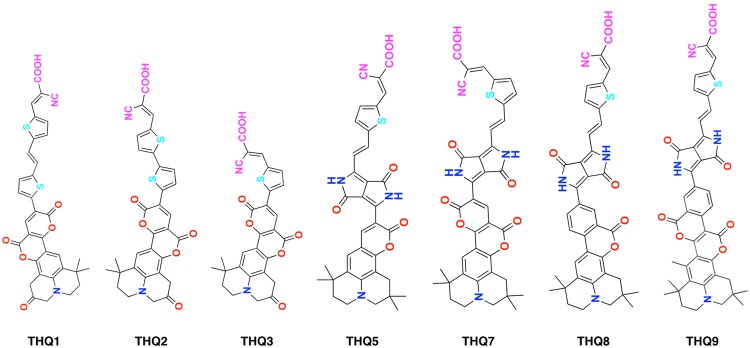
The sketch of all the investigated Tetrahydroquinoline dyes.

The seven lead dyes from the THQ family studied by us are represented in Fig. 1. The structural and electronic properties of the designed THQ dyes were computed in gas and solvent phase to get deep insight into investigated phenomena. After the photo-excitation of dye in DSSCs, *J*_*SC*_ and *V*_*OC*_ are the most relevant parameters to calculate theoretically with precision to compare its performance as an efficient sensitizer. Electron injection mainly depends on the absorption capacity of photon, ground state oxidation potential and optimum HOMO-LUMO energy gap of the respective dye. Parameter like *J*_*SC*_ can be estimated in terms of Δ*G*_*inject*_ and the computed critical energy parameters^25^ of the present study are tabulated in Table 1 for B3LYP/6–31 g(d, p) level of theory in gas phase and with TD CAM-B3LYP/6–31 g(d, p) level of theory in acetonitrile solvent (Applied equations for computation are discussed in Methods section).

**Table 1:** Estimated critical energy parameters (in eV) and electron injection time (in ns) with B3LYP/6–31 g(d, p) level of theory in gas phase.

Name	Gas Phase											Acetonitrile		
	*E*_*HOMO*_	*E*_*LUMO*_	*λ*_*max*_	*E*_*Ex*_^dye*^	*ΔG*_*inject*_	*λ*_*h*_	*λ*_*e*_	*λ*_*total*_	*V*_*OC*_	*ΔG*_*reg*_	τ(ns)	*q*_*CT*_	*D*_*CT*_	*S*
THQ1	−5.40	−3.03	2.58	2.82	−1.18	0.31	0.30	0.61	0.97	−10.2	1.42	0.593	2.842	0.963
THQ2	−5.54	−3.02	2.68	2.86	−1.14	0.31	0.30	0.61	0.98	−10.3	1.59	0.581	2.646	0.964
THQ3	−5.80	−3.05	2.87	2.97	−1.03	0.30	0.35	0.65	0.95	−10.6	1.87	0.571	2.525	0.959
THQ5	−4.99	−3.17	2.07	2.92	−1.08	0.28	0.30	0.58	0.83	−9.79	3.42	0.540	3.226	0.957
THQ7	−5.15	−3.32	2.08	3.07	−0.93	0.31	0.28	0.59	0.68	−9.95	3.06	0.552	2.277	0.970
THQ8	−5.14	−3.35	2.19	2.95	−1.05	0.19	0.29	0.48	0.65	−9.94	2.38	0.551	3.538	0.962
THQ9	−5.28	−3.40	2.23	3.05	−0.95	0.20	0.28	0.48	0.60	−10.9	2.61	0.585	2.517	0.971
DI293	−5.07	−2.81	2.58	2.49	−1.51	0.28	0.32	0.60	1.19	−9.87	1.64	0.610	4.042	0.944

Computed CT parameters (DCT in Å and qCT in |e-|) in acetonitrile solvent with TD CAM-B3LYP/6–31 g(d, p) level of theory.

The obtained values of Δ*G*_*inject*_ for all of the cases are negative which implies the spontaneity of the electron injection process. Also, Islam *et al*. proved that the injection efficiency of the electrons in the excited state (*Φ*_*inject*_) tends to approach 1 when Δ*G*_*inject*_ is greater than 0.20eV^26^. THQ1 (−1.18 eV) is showing the highest efficiency while THQ7 (−0.93 eV) displays the lowest one. The sequence of dyes considering Δ*G*_*inject*_ is: THQ1 > THQ2 > THQ5 > THQ8 > THQ3 > THQ9 > THQ7. Comparable assessments of dyes regeneration efficiency have been calculated (See equation in methods section) and the computed values of Δ*G*_*regn*_ of all the dyes are negative which implies the feasibility of the process. The values of Δ*G*_*regn*_ vary from 9.79 to 10.9 eV that is much higher than the accepted value of Δ*G*_*inject*_ and confirming that the dye regeneration process is more favorable compared to electron injection^27^. The THQ9 with the lowest Δ*G*_*regn*_ value suggests that it will minimize the undesirable charge recombination between oxidized dye and photoexcited electrons of the dye molecule efficiently, due to the presence of diketopyrroleopyrrole (DPP) unit with one benzene ring.

The open circuit voltage (*V*_*OC*_) that can be expressed^28^ by the Equation (1), depends on the recombination of the dye with electrolyte. This type of recombination could provide insufficient blocking effect due to relatively low adsorption density of dyes^29^. Thus to enhance the effect, the shape and structures of the dye need to be optimized. Ning *et al*. suggested that upshifting of the sensitizer *E*_*LUMO*_ will increase the *V*_*OC*_ and reduce the charge recombination^30^. Our computed results showed that THQ2 has highest while THQ9 has lowest *V*_*OC*_ value among the studied dyes. Analyzing individual dye structures, we can firmly state that presence of DPP unit is responsible for lowering *V*_*OC*_ values for THQ5, THQ7-THQ9^31,32^ and absence of DPP fragments in THQ1-THQ3 resulted in higher *V*_*OC*_ values.

1 $${V}_{OC}={E}_{LUMO}-{E}_{CB}^{Ti{O}_{2}}$$

**Figure 2:**
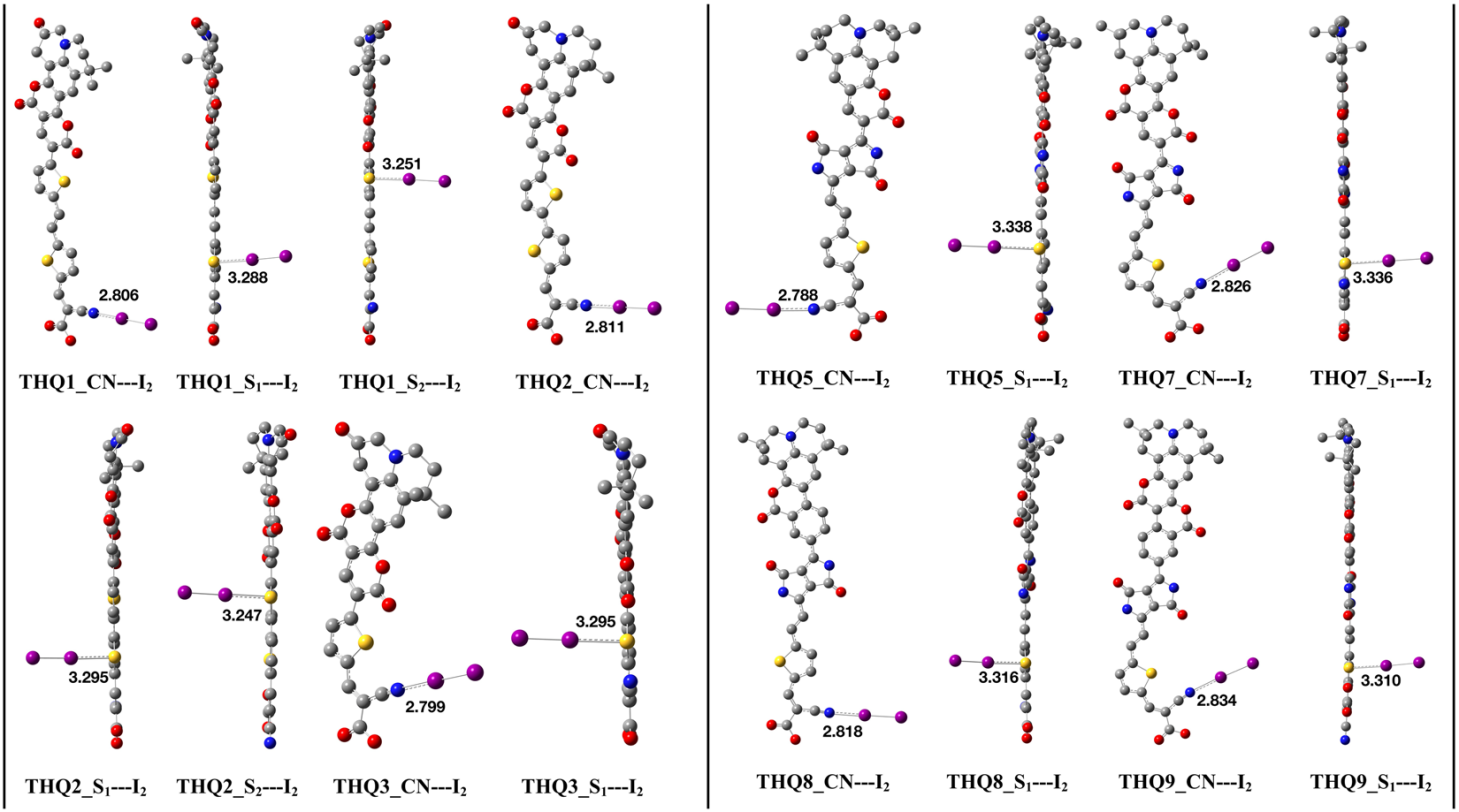
Optimized structures of all isolated dye-I_2_ complex. Color code: Gray, Yellow, Red, Blue and Purple representing C, S, O, N, and I atoms.

Electron recombination is one of the most important factor which reduce *V*_*OC*_ by two ways: (i) redox couple of electrolytes, and ii) oxidized dyes^2^. Richards *et al*.^33^ established that electron recombination to the electrolyte dominated by the free iodine concentrations and thus I_2_ is more inclined to bind with heteroatoms (like O, N, S etc.) of dye molecules *via* non-covalent halogen bond interaction. To estimate the electron recombination on TiO_2_ surface, we considered three different configurations of the isolated-dye…I_2_ complex. Three configurations are: (a) I_2_ (I_A_ − I_B_) binds with the N of CN moiety; (b) S atom of thiophene ring (S_1_) near acceptor units binds with I_2_ (I_C_ − I_D_) atom; and (c) S atom of thiophene ring (S_2_) far form acceptor units binds with I_2_ (I_E_-I_F_) atom. The optimized structures of dye…I_2_ complex for all the seven dyes are depicted in Fig. 2 while the bond lengths of I-X (X = I, N, S) are listed in Table S1 (supplementary material). In dye…I_2_ complex, intramolecular I-I bond lengths are larger than the covalent radii (2.86 Å), which imply the iodine dimers are weekly bound and the distance of CN and iodine (CN–I_A_) atom is smaller than the net van der Waals radii 3.53 Å but larger than the net covalent radii 2.03 Å. Also, the bond lengths between thiophene S and iodine (S–I_C/E_)are larger than the covalent radii, while smaller than the net van der Waals radii (3.78 Å) of the binding atoms. Similar outcomes was also observed by Xu *et al*.^34^.

The trend of CN–I_A_ bond lengths is: THQ9 > THQ7 > THQ8 > THQ2 > THQ1 > THQ3 > THQ5 while the length of I_A_-I_B_ is following: THQ5 > THQ3 > THQ1 > THQ2 > THQ8 > THQ7 > THQ9. Moreover, the sequence of S_1_–I_C_ bond length (except THQ1 and THQ2) is THQ5 > THQ7 > THQ8 > THQ9 > THQ3 and the trend for I_C_-I_D_ is: THQ3 > THQ9 > THQ7 > THQ8 > THQ5. In both cases, the interaction of dye and iodine is increasing with the increase of separation distances of iodine. On the other hand, THQ1 and THQ2 are showing different trend in case of S_2_–I_E_ bonds. It might be possible due to the presence of two different thiophene rings, as strongly supported by literature^34^. Based on above analysis, it is quite difficult to choose which dye will transfer the I_2_ away from the semiconductor (TiO_2_) surface and help to reduce the charge recombination by inhibiting the local concentration of I_2_ on TiO_2_.

Built-in push-pull structure in the designed dyes possesses significant charge separation that confirms the high overall efficiency of photo-to-electron conversion^35^. Index of spatial extent method is used to describe the ICT process qualitatively. Spatial index including distance between two barycenters of the density depletion and density accumulation distribution upon excitation (*D*_*CT*_) in Å, transferred charge (*q*_*CT*_) in e, and overlap integral (*S*) are reported in Table 1. Analysis of data suggested that chromophore in dye with larger overlap integral increases the amount of charge transfer with longer *D*_*CT*_ which confirms proper charge separation of donor and acceptor moieties^35^. The predicted trend of *S* is as following: THQ9 > THQ7 > THQ8 > THQ2 > THQ1 > THQ3 > THQ5, while *D*_*CT*_ displays the following tendency: THQ7 < THQ9 < THQ3 < THQ2 < THQ1 < THQ5 < THQ8. The increased value of *S* implies that the addition of DPP unit as an additional donor and acceptor moiety makes the smooth flows of electron transfer from the ground state to excited state. However, from the above sequence, it is not possible to select which lead dye will be more efficient for DSSCs based on ICT. Again, upon the analysis of the amount of charge transferred, the *q*_*CT*_ sequence of dyes is as following: THQ1 > THQ9 > THQ2 > THQ3 > THQ7 = THQ8 > THQ5. There are some literature evidences that also claimed advantages of D_CT_ and S which may reduce the charge recombination of the sensitizers, besides excellent charge separation^34,36^. If one investigates the Fig. S1 reported in Supplementary material, the overlap integral space can be visualized clearly upon electronic excitation. The blue and green colors indicate the centroid of hole (*C*+) or donor and the centroid of electron (*C−*) or acceptor.

Furthermore, all designed dyes had been checked based on the Marcus theory^37^ by calculations of the charge transfer rate using the following equation:

2 $${k}_{ET}=\frac{1}{\sqrt{{\lambda }_{total}}}\sqrt{\frac{\pi }{{\hslash }^{2}{k}_{B}T}}|{V}^{2}|\exp \{-\frac{{\lambda }_{total}}{4{k}_{B}T}\}$$

In equation (2) except reorganization energy, *λ*_*total*_, all the parameters on the right-hand side are constant. Thus electron transfer rate constant (*k*_*ET*_) depends only on *λ*_*total*_, which is the sum of hole reorganization (*λ*_*h*_) and electron reorganization (*λ*_*e*_) energy, whose values can be estimated by the Equations (3) and (4), respectively.

3 $${\lambda }_{h}=({E}_{0}^{+}-{E}_{+}^{+})+({E}_{+}^{0}-{E}_{0}^{0})$$

4 $${\lambda }_{e}=({E}_{0}^{-}-{E}_{-}^{-})+({E}_{-}^{0}-{E}_{0}^{0})$$

where, ${E}_{0}^{+}\,({E}_{0}^{-})$ denotes the energy of cation (anion) calculated from the optimized structure of the neutral molecule; ${E}_{+}^{+}\,({E}_{-}^{-})$ is the energy of cation (anion) calculated from the optimized cation (anion); ${E}_{+}^{0}\,({E}_{-}^{0})$ is the energy of neutral molecule computed from the cationic (anionic) state; and ${E}_{0}^{0}$ is the energy of neutral molecule at the ground state.

Difference between the *λ*_*h*_ and *λ*_*e*_ is also important to determine the equilibrium properties for hole- and electron transport while smaller *λ*_*total*_ accelerates the charge-carrier transport rates and *vice-versa*. The value of calculated *λ*_*total*_ is found within a range of 0.48 eV to 0.65 eV with following trend: THQ9 = THQ8 < THQ5 < THQ7 < THQ1 = THQ2 < THQ3. Also, the lifetime (${\rm{\tau }}$) of first excited state (*S1*) is a crucial factor to modulate the charge transfer phenomenon efficiently. This can be estimated by the relationship from Chaitanya *et al*.^38^ $\tau =1.499/(f{E}^{2})$; where *E (cm*^−*1*^*)* is the excitation energy for *S1* and *f* is the oscillator strength for the respective state. The dye with a more extended lifetime in the first excited state is predicted to transfer charge efficiently^39^. Our calculations of charge transfer shows the following trend: THQ5 (3.42 ns) > THQ7 > THQ9 > THQ8 > THQ3 > THQ2 > THQ1 (1.42 ns). The difference between the redox potential and the *E*_*HOMO*_^13^, as well the energy gap of the sensitizer affect the lifetime to reduce undesirable reaction like charge recombination^40^. On the basis of Marcus theory and lifetime in the excited state, we can preliminary comment that among the seven designed lead dyes THQ5, THQ7, THQ8, andTHQ9 are more proficient than THQ1, THQ2, and THQ3.

Based on the above analysis, we can firmly confirm that all of the designed dyes have the desirable optoelectronic properties including electron injection and lifetime in the excited state, open circuit voltage, ICT parameters, and regeneration of the dye itself. However, we are not able to conclusively comment on the overall performance because the effectiveness of isolated dye is not the only factor to determine the efficiency of DSSCs. The adsorption characteristic of the dye, the interface of dye@TiO_2_ and charge transfer phenomenon are also important factors to determine the efficiency of DSSCs, which are computed and thoroughly discussed in the next section.

### Structural and electronic properties of dye-TiO2 system

To elucidate the stability and interfacial charge transfer phenomenon of the designed THQ dyes on anatase TiO_2_ (101) surface, we have calculated the adsorption energy, 3D charge density difference (CDD) plot, Bader charge analysis, planar averaged electrostatic potential and CDD with respect to Z-direction, and partial density of states (PDOS) using first principle approach.

**Figure 3:**
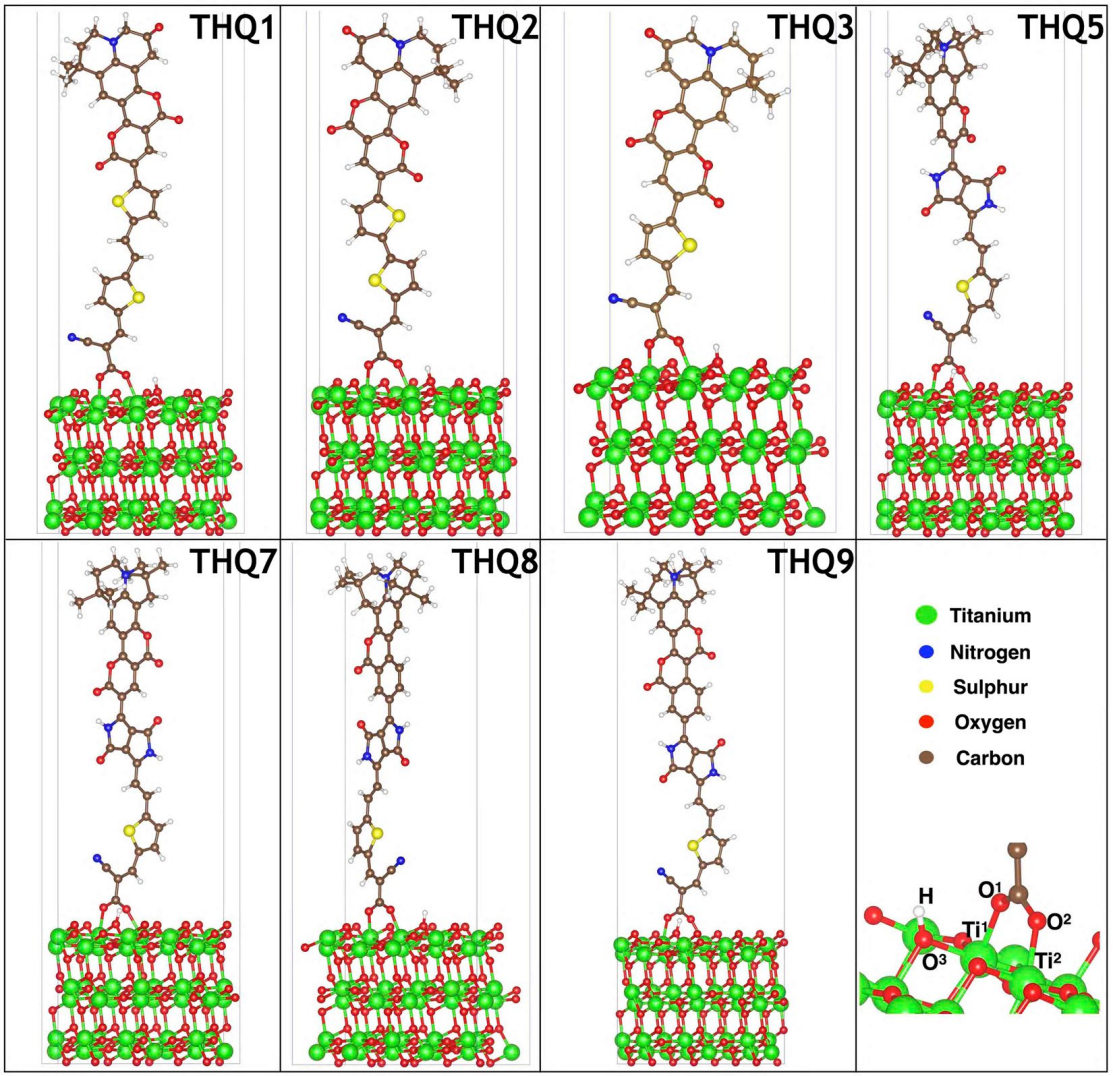
Optimized by DFT + U method with optB86b-vdW density functional stable geometrical structures of studied dye@TiO_2_ systems.

Adsorption energy varied with different binding modes of anchoring group of dye and TiO_2_ surface. In literature, three different types of binding such as monodentate, chelated and bridged bidentate^41^ are prevailed and later one being the most stable^12,23,42^. Thus in the present study, the equilibrium interface geometry and adsorption energy of the dyes on anatase TiO_2_(101) surface were calculated based on the bridged bidentate bonding. Optimized configurations of the interface for all the dyes are illustrated in Fig. 3. It is notable to mention that the adsorption energy, charge density, and Bader charge analysis had been computed based on the deprotonated dye (negatively charged) and protonated TiO_2_ (101) surface (positively charged)^43,44^. Geometrical parameters and adsorption energy for the different systems were calculated and reported in Table 2. Adsorption energy is one of the measures to describe the strength of interaction or electronic coupling between TiO_2_ surface and a dye. In our study, THQ9 is showing highest adsorption energy (−3.77 eV) whereas THQ5 having the least (−1.42 eV) energy. The adsorption energy is calculated according to the Equation (5):

5 $${\rm{\Delta }}{E}_{ads}={E}_{dye+Ti{O}_{2}}-[{E}_{dye}+{E}_{Ti{O}_{2}}]$$

where, *ΔE*_*ads*_*, E*_*dye+TiO2*_*, E*_*dye*_*, E*_*dye+TiO2*_ are the adsorption energy, energy of the system, free dye energy and TiO_2_(101) slab energy, respectively. The adsorption energy has been computed by the equation (5) with the use of van der Waals density functional, optB86b-vdW, implementing Hubbard corrections (Table 2). The average bond length between Ti and O of the –COOH group is approximately the same value for all of the systems^42^. Plane averaged electrostatic potential of TiO_2_ slab is lower than that of the dye molecule, suggesting charge transfers from dye molecule to TiO_2_, in line with results reported in Table 2. The electrostatic potential differences between dye and TiO_2_ slab also has a good agreement with the Bader charge transfer. The efficiency of DSSCs highly relies on both the rate of electron injection and the efficient regeneration of dye. Not only that, but also the HOMO and LUMO levels of the dye sensitizers are lower and higher enough than the redox couple and TiO_2_ conduction band, respectively, as reported in Table 1.

**Table 2:** Calculated adsorption energy (in eV) and bond lengths (in Å) of the most stable structure of dye@TiO2 systems by DFT + U method with optB86b-vdW density functional.

System	*E*_Ads_	*d*_Ti_^1^_-O_^1^	*d*_Ti_^2^_-O_^2^	*d*_Ti-O_	*d*_H-O_
THQ1/TiO_2_	−2.19	2.12	2.05	2.09	0.974
THQ2/ TiO_2_	−2.03	2.09	2.07	2.09	0.974
THQ3/TiO_2_	−1.77	2.13	2.06	2.10	0.973
THQ5/TiO_2_	−1.42	2.09	2.06	2.08	0.973
THQ7/TiO_2_	−1.92	2.10	2.06	2.08	0.973
THQ8/TiO_2_	−2.01	2.09	2.06	2.06	0.973
THQ9/TiO_2_	−3.77	2.10	2.06	2.08	0.973
DI293/TiO2	−2.12	2.09	2.05	2.07	0.974

**Figure 4:**
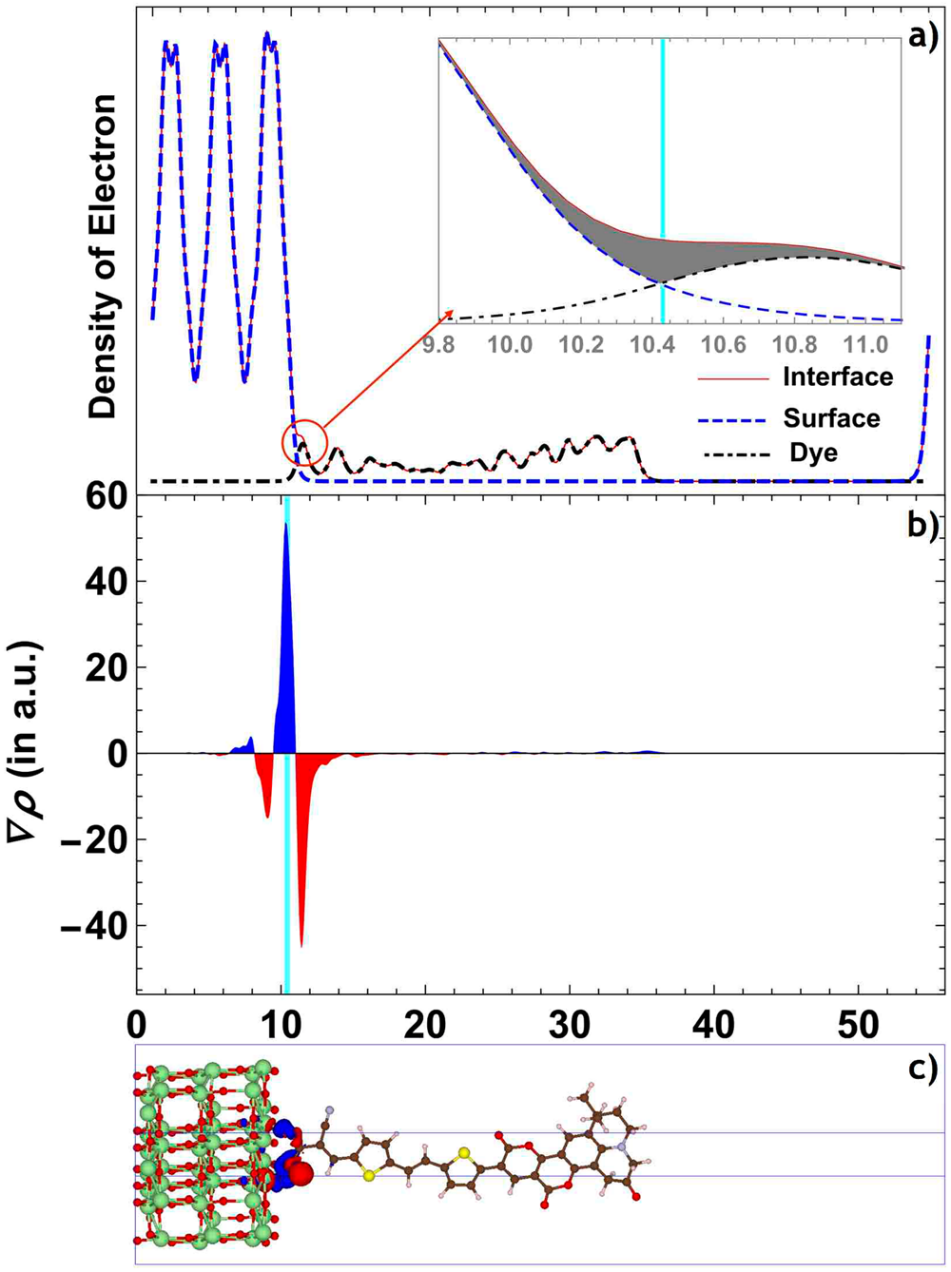
For THQ1 (**a**) Planar averaged charge density of dye@TiO_2_ system after absorption (red), isolated dye (dashed) and surface (dotted) at the same relative positions. It is clear that there is no significant charge redistribution away from the interface region. Inset: magnifying the interface region and yellow part showing the amount of injected charge. (**b**) Planar average charge density difference as a function of position in the Z-direction, in Å. (**c**) 3D charge density difference with an isovalue of 0.006e/Å^3^. Blue and red color represents charge accumulation and depletion in space. The vertical cyan line indicates the interface line of dye@TiO2 system.

Next, to gain insight into the dye-surface interactions, we have examined the charge redistribution between the dye and surface after and before the adsorption of dye at the same relative ionic positions and have shown the results in the Fig. 4(a). Here we have only discussed THQ1 dye, which is similar to other systems. Evaluating the figure, it is clear that there is definite charge injection from the dye molecule to the interface. For the better understanding, we consider the charge-density difference and the average of this quantity over the (x, y) plane is depicted in Fig. 4(b) suggesting that the charge at the interface originates from the dye molecule. This phenomenon is also confirmed through the 3D charge density difference plot illustrated in Fig. 4(c). Bader charge analysis^45,46^ results are illustrated in Table 3. This indicates that the charge transfer from the dye to interface region originates from the dye. The accumulation of charge at the interface is also in line with the trend mentioned above, except for THQ7, which comes after THQ9 but above THQ5 and THQ8. When rate of charge transfer reaches equilibrium state, net charge collection at interface leads to the formation of built-in electric field and helps to continuous electron injection from the dye to interface and interface to the TiO_2_ surface after the dye excitation. Quite similar results are obtained from analysis for other dyes as illustrated in Figs S2–S7 in Supplementary material file.

**Table 3:** *Ab initio* (VASP) calculated band gaps (in eV) after adsorption of the Dye/TiO_2_ system and Bader charge (in e^−^). *d*_*gap*_ (in eV) indicates the difference before and after adsorption.

System	*E*_gap_	*d*_gap_	Bader charge
TiO_2_(101)	1.70	NA	NA
THQ1/TiO_2_	0.90	0.80	0.27
THQ2/ TiO_2_	1.01	0.69	0.31
THQ3/TiO_2_	1.25	0.45	0.28
THQ5/TiO_2_	0.53	1.17	0.36
THQ7/TiO_2_	0.40	1.30	0.33
THQ8/TiO_2_	0.40	1.30	0.16
THQ9/TiO_2_	0.42	1.70	0.36

**Figure 5:**
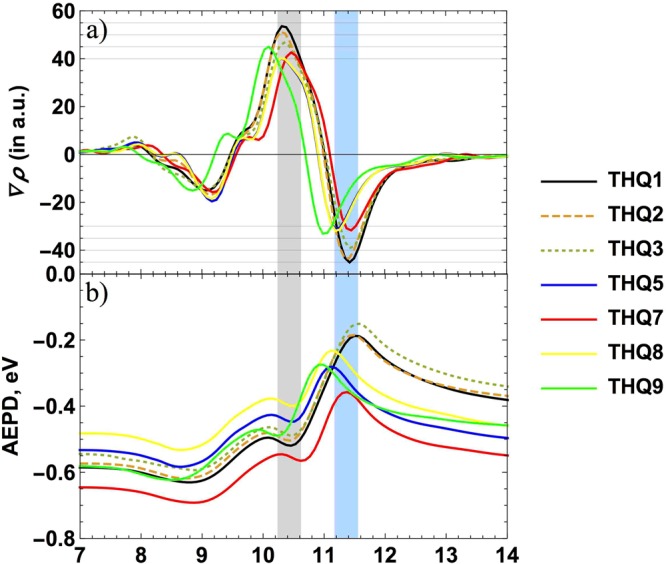
(**a**) Comparison of planar averaged charge density differences and (**b**) planar averaged electrostatic potential differences as a function of position in the Z-direction (in Å) of all different dyes. Grey and cyan shaded stripes are representing the approximate position of the interface and –COOH group of dye, respectively.

The differences of planar average charge density difference and planar average electrostatic potential differences are plotted in Fig. 5 with respect to *Z-*direction. The Fig. 5(a) depicts the important features of the interface in order to compare the electron injection efficiency of the dye. Among seven dyes, charge accumulation at interface of THQ1 is the highest while THQ8 showing the least of charge accumulation. The trend of accumulation is as following: THQ1 > THQ2 > THQ3 > THQ9 > THQ5 = THQ7 > THQ8. It is important to mention that THQ1 and THQ2 possess the largest amount of charge accumulation at interface as well as the highest free energy change for electron injection efficiency (see Table 1). The Fig. 5(b) represents the planar average self-consistent electrostatic potential difference as a function of the position in the Z-direction. Potential for all three components are dipole corrected. At interface the formation of well-shaped potential-well explains the charge transfer phenomenon that is in line with the previous discussions.

**Figure 6:**
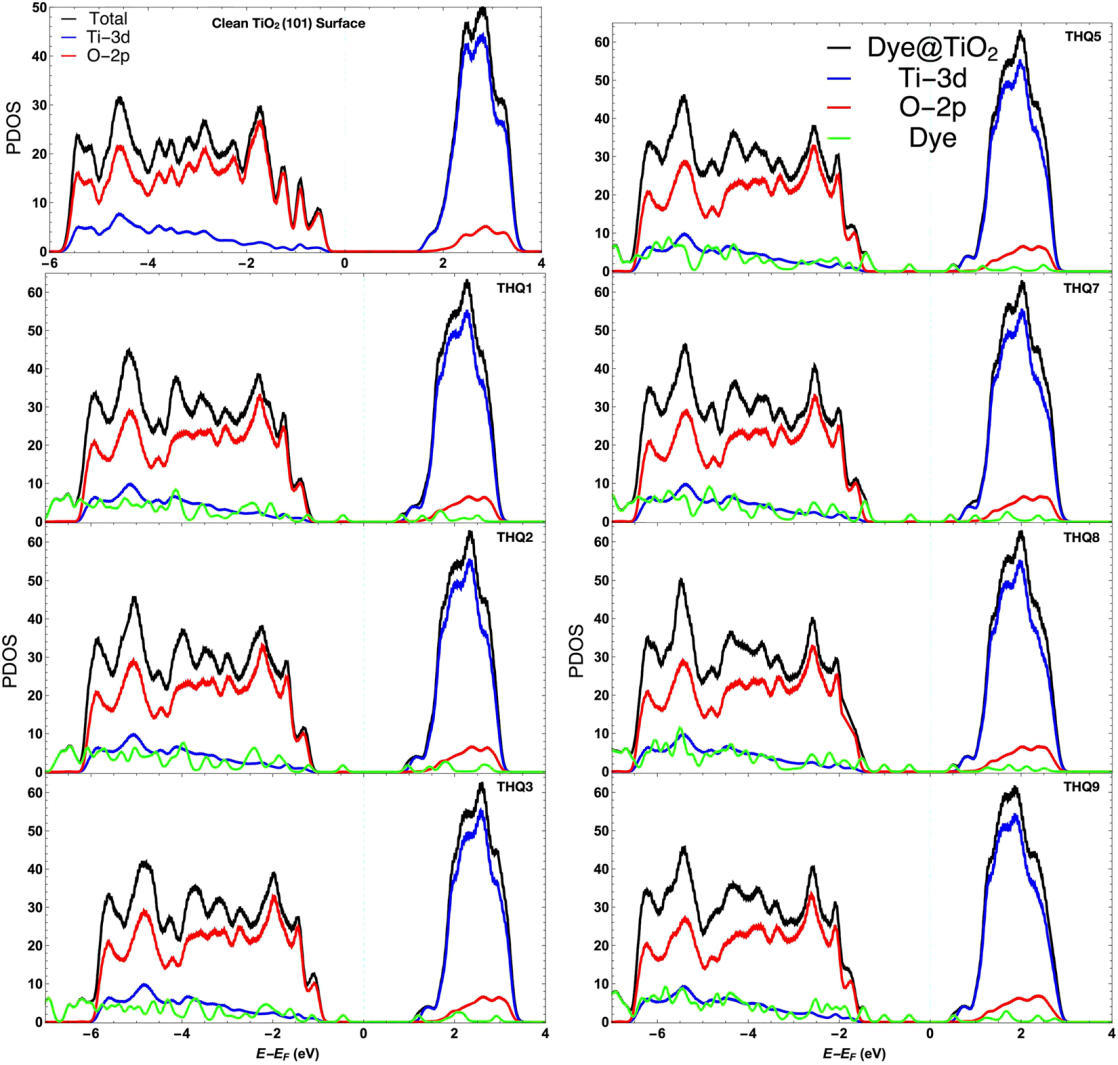
The partial density of states (PDOS) of adsorbed complexes at different interfaces. The vertical dashed line indicates that the Fermi level is set at zero energy.

To further elucidate the electronic properties/band alignment of the dye@TiO_2_ interfaces, it is insightful to analyze the PDOS of adsorbed complexes. PDOS of the different interfaces is displayed in Fig. 6. After the adsorption of dye, for all considered systems, the conduction band minima (CBM) and valence band maximum (VBM) of TiO_2_ showed upward shift relative to Fermi level. After adsorption, the band gap of the complexes is reduced due to the introduction of sharp occupied molecular energy levels in the band gap regions from the dye. It is supported by the facts that narrow band-gap extends the absorption region and controls the photo adsorption efficiency while the energy difference between ${E}_{CBM}^{Ti{O}_{2}}$ and *E*_*LUMO*_ of the dye regulates injection efficiency or short circuit current density. The computed band gap of TiO_2_(101) surface decreased from 1.70 eV to 0.40 eV (THQ7/TiO2, THQ8/TiO_2_) for dye@TiO_2_ system; while the range is 0.40 (THQ7/TiO2, THQ8/TiO_2_) to 1.25 eV (THQ3/TiO2) for all studied composite Dye@TiO_2_ systems. Band gaps for THQ1, THQ2, THQ3, THQ5, and THQ9 are 0.90 eV, 1.01 eV, 1.25 eV, 0.53 eV and 0.42 eV, respectively. The narrow band gap of specific dyes can be explained due to the lower order occupied orbitals of the dyes. Thus this characteristic helped in moving the conduction band towards lower energy. For all the cases, LUMO of the adsorbed dyes is coupled with the CBM of TiO_2_ surface that allows intermolecular electrons to be excited to the surface and increases the overlap between the LUMO and CBM, improving the electron injection process. Also, inner LUMO of the dye expedites electron injection process. In Fig. 6, it can be confirmed that the DOS of the dye has a strong overlap with the valence band of the TiO_2_ semiconductor, which means that dye introduces few pi-occupied levels on top of the TiO_2_ valence band. The newly pi-occupied levels from the dye become the new top-part of the valence region. In case of DI293 (highest experimental %PCE dye from our model dataset^13^), the band gap is 1.0 eV (see Fig. S8 in Supplementary material file). Moreover, the DOS of adsorbed dye molecules shift to higher energy (compared to before adsorption), illustrated a significant amount of charge transfer from dye to TiO_2_ surface. This is because of the HOMO of dye shifted to band gap regions while LUMO shifted toward the conduction band about 0.18–0.58 eV (Table S2 reported in Supplementary material). Based on PDOS, it is crucial to highlight that, our designed dyes (except THQ2 and THQ3) have lower band gap than DI293 with highest photo-conversion efficiency value obtained in modeling study, suggesting that their photon absorption efficiency would be higher than that of the DI293. Insertion of DPP^47^ moiety, also with the double bonded oxygen as an additional donor and acceptor, enhances the photovoltaic efficiency of the designed dye. The above results strongly support the conclusions of our previous study13.

## Discussions

In summary, we have performed systematic, comprehensive investigations to evaluate seven ‘lead THQ dyes’. These dyes were selected by our group in previous study as characterized by high-predicted %PCE. The current work was carried out to reveal the relation between dyes’ structure and their photo-conversion efficiency. The optic-electronic properties of the isolated dyes were computed by DFT and TD-DFT with solvent effect, whereas necessary. Interfacial properties of the adsorbed dye on TiO_2_ (101) surface was investigated with the use of first principle approach with van der Waals density functional, in conjunction with on-site Coulomb interaction for Ti 3d orbital. Outcomes from the first step can be summarized as follows: THQ9 emerged among the top three dyes concerning charge transfer characteristics (*S, τ, qCT, λ*_*total*_) while giving lower value for open circuit voltage (*V*_*OC*_), and electron injection energy (*ΔG*_*inject*_) than other considered dyes. Furthermore, three different binding modes and their structural attributes of dye–I_2_ complex are evaluated to address electron recombination process. Analyzing dye regeneration efficiency, THQ3 shows the highest value. Based on calculations for the isolated dyes, it can be concluded that all of the considered dyes possess potential properties to become an efficient dye-sensitizer for DSSCs. Introspecting the geometrical interface structures, PDOS, planar averaged charge density and potential energy difference of dye@TiO_2_ system; we infer that there is robust electronic coupling between all dyes and TiO_2_ surface. The PCEs of THQ7, THQ8, and THQ9 dyes are better than for the other four dyes when dye@TiO_2_ are considered, due to their lowest adsorption energy, lower band gap and higher Bader charge. Interestingly, the other four dyes also possess all required electrochemical parameters to act as efficient and improved dye sensitizers for DSSCs, compared to the products available on the market. Therefore, the insertions of double bonded oxygen to the aromatic carbon certainly improve the photovoltaic performance and energy conversion efficiency of sensitizers. In conclusion, based on the comprehensive analysis, modeling and design followed by the structural and electronic properties evaluation of the lead dyes, we proposed them as efficient, potential sensitizers for future DSSCs with improved PCE.

## Methods

Geometry optimization and estimation of all photophysical parameters of the isolated dyes were performed in Gaussian09 package^48^. Ground state and excited state calculations were done with B3LYP and CAM-B3LYP functional within DFT and TD-DFT framework, respectively. It is notable to mention that CAM-B3LYP functional for the TD-DFT method provides reasonable prediction for the excitation energies and the absorption spectra due to its long-range coulomb-attenuating method^5,13,49^. In all the cases, 6–31 g(d,p) basis set is used for C, H, O, N, and S atoms while LANL2DZ effective core potential (ECP) for I atoms. For the ICT parameters and to visualize centroids calculations were performed by code Multiwfn 3.3.9^50^. The overall efficiency of photo-to-electron conversion in DSSCs is determined by the integral of short-circuit photocurrent density (*J*_*SC*_), open–circuit photo voltage (*Voc*), fill factor (FF) and incident solar power on the cell^1^:

6 $$\eta =FF\frac{{J}_{SC}\times {V}_{OC}}{{P}_{IN}}\times 100 \% $$

The *J*_*SC*_ depends on the absorption coefficient of the dye and the interaction between the dye and the nanocrystalline TiO_2_ surface. It can be computed by the following equation:

7 $${J}_{SC}=\int LHE(\lambda ){{\rm{\Phi }}}_{inject}{\eta }_{collect}d\lambda $$

where *LHE* is light harvesting efficiency at wavelength *λ*, *Φ*_*injec*t_ is quantum yield for electron injection efficiency and *η*_*collect*_ is the charge collection efficiency which only depends on the architecture of DSSCs while other parameters strongly depend on the structural and quantum phenomenon of the dye. Katoh *et al*.^51^ related injection efficiency to the free energy change for the electron injection (*ΔG*_*inject*_) that can be defined through following equation:

8 $${\rm{\Delta }}{G}_{inject}={E}_{ox}^{dy{e}^{\ast }}-{E}_{CB}^{Ti{O}_{2}}$$

where ${E}_{ox}^{dy{e}^{\ast }}$ is the oxidation potential of the dye in the excited state, and ${E}_{CB}^{Ti{O}_{2}}$ is the reduction potential of the conduction band of semiconductor (TiO_2_). The ${E}_{ox}^{dy{e}^{\ast }}$ can be computed by subtracting absorption energy associated with the maximum wavelength (λ_max_) from the ground state oxidation potential (${E}_{ox}^{dye}$) of the dye that is equal to the negative value of the HOMO energy of the isolated dyes.

9 $${E}_{ox}^{dy{e}^{\ast }}={E}_{ox}^{dye}-{\lambda }_{max}$$

Also, dye regeneration efficiency can be measured by the driving force of regeneration (Δ*G*_*regn*_) between electrolyte and oxidized dye *via* the following expression:

10 $${\rm{\Delta }}{G}_{regn}={E}^{redox}-{E}_{ox}^{dye}$$

Calculations of TiO_2_ surface and the interface of dye@TiO_2_ were performed using the Vienna *ab initio* Simulation Package (VASP)^52–54^ code under generalized gradient approximation (GGA). To account for the van der Waal (vdW) interactions, non-local density functional like vdW density functional, optB86b-vdW^55^, was employed as implemented in VASP by J. Klimeš *et al*.^56,57^. A Hubbard onsite Coulomb repulsion term^58^ was added (DFT + U) to improve the description of the on-site Coulomb interaction in the Ti-d states with an effective U parameter. It is crucial to choose effective U parameter, as the band gap of anatase is directly proportional to the U parameter with the overestimation of lattice description^59^. Our study also follows the same trend. Based on the previous work^60^ by our group and Paes *et. al*.^61^ study we choose effective U value of 3.5 eV.

**Figure 7:**
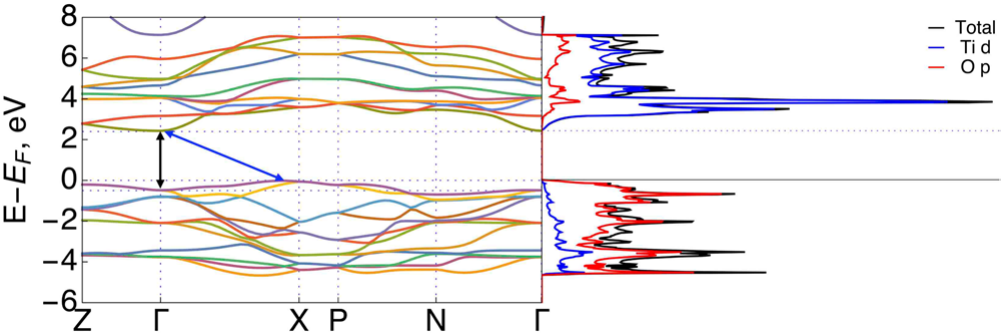
Band diagram and PDOS of bulk TiO2 with an indirect and direct band gap of anatase. The distance between the top of the valence band at X and the bottom of the conduction band at Γ, amounts to 2.4 eV (double ended blue arrow) and from Γ to Γ is 2.9 eV (double ended black arrow) represent the indirect and direct band gap, respectively.

In the dye@TiO_2_ system, we describe C-1s^2^, O-1s^2^, N-1s^2^, Ti-[Ne]3s^2^ and S-[Ne] core electrons with the projector augmented wave (PAW) pseudopotentials^62^. The cutoff energy of the plane-wave basis set was 400 eV with a Γ centered Monkhorst-Pack grid of 0.03 Å^−1^ separation (equivalent to a k-points mesh of 8 × 8 × 11, 2 × 2 × 1 for bulk anatase, and TiO_2_(101)-c(4 × 2) supercells, respectively). The total energy converged to <1 meV/atom. All the structures considered in this study were fully relaxed until all the forces and energy were less than 0.025 eV/Å and 10^−6^ eV/atom, respectively. The DFT + U computed optimum lattice parameter of bulk anatase TiO_2_ were a = 3.85 Å and c/a = 2.52 Å; while the indirect band gap is lower in the order of 2.4 eV (direct band gap 2.9 eV) compared to experimental value of 3.2 eV. The band structure, corresponding Brillouin zone with direct and indirect band e gap are shown in Fig. 7. The anatase TiO_2_(101) surface was modeled with three O-Ti-O layers along the [001] direction and 15 Å vacuum thickness between slabs resulting in approximately 25 Å dimension in [001] direction.

### Data Availability Statement

The results of calculations can be obtained by request from the authors.

## Electronic supplementary material

Supplementory information
