# Supplementary material for: Insight into the optoelectronic properties of designed solar cells efficient tetrahydroquinoline dye-sensitizers on TiO2(101) surface: first principles approach
Source: Sci Rep. 2018 Jul 20;8:10997. doi: 10.1038/s41598-018-29368-9 (PMC6054641; doi:10.1038/s41598-018-29368-9)
Supplement: Supplementary file 1 — Supplementory information [file 41598_2018_29368_MOESM1_ESM.docx]

**Insight into the optoelectronic properties of designed solar cells efficient tetrahydroquinoline dye-sensitizers on TiO_2_(101) surface: first principles approach**

Juganta K. Roy^1^, Supratik Kar^1^, Jerzy Leszczynski^1*^

^1^Interdisciplinary Center for Nanotoxicity,

Department of Chemistry, Physics and Atmospheric Sciences,

Jackson State University, Jackson, MS-39217, USA

*Corresponding author

Prof. Jerzy Leszczynski, Phone: +1 601 979 3723; fax: +1 601 979 7823; E-mail: [jerzy@icnanotox.org](mailto:jerzy@icnanotox.org)

**Figure S1**: Graphical representation of CT which associated centroids of hole and electron (C+/C-). Computed difference in total density from S0 to S1 for all the dyes with isovalue 0.00045 a.u. performed in acetonitrile solvent using CAM-B3LYP function together with 6-31-g (d, p) basis set.





**Figure S2-S7:**(a) Planar averaged charge density of dye@TiO2 system after absorption (red), isolated dye (dashed) and surface (dotted) at the same relative positions. It is clear that there is no significant charge redistribution away from the interface region. Inset: magnifying the interface region and yellow part showing the amount of injected charge. (b) Planar average charge density difference as a function of position in the Z-direction. (c) 3D charge density difference with an isovalue of 0.006e/Å^3^. Blue and red color represents charge accumulation and depletion in space. The vertical cyan line indicates the interface line of dye@TiO2 system.





**Figure S2: For THQ2**


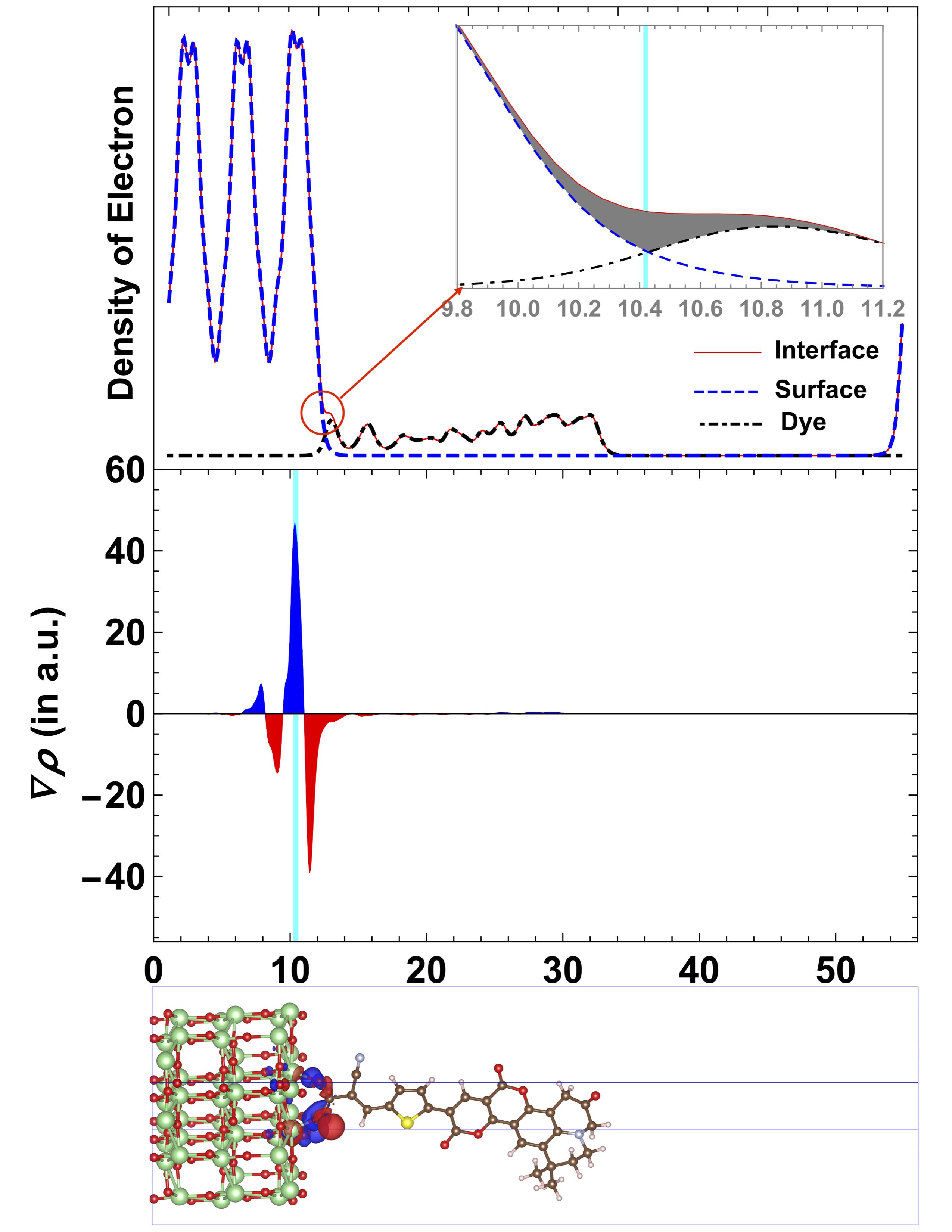


**Figure S3: For THQ3**


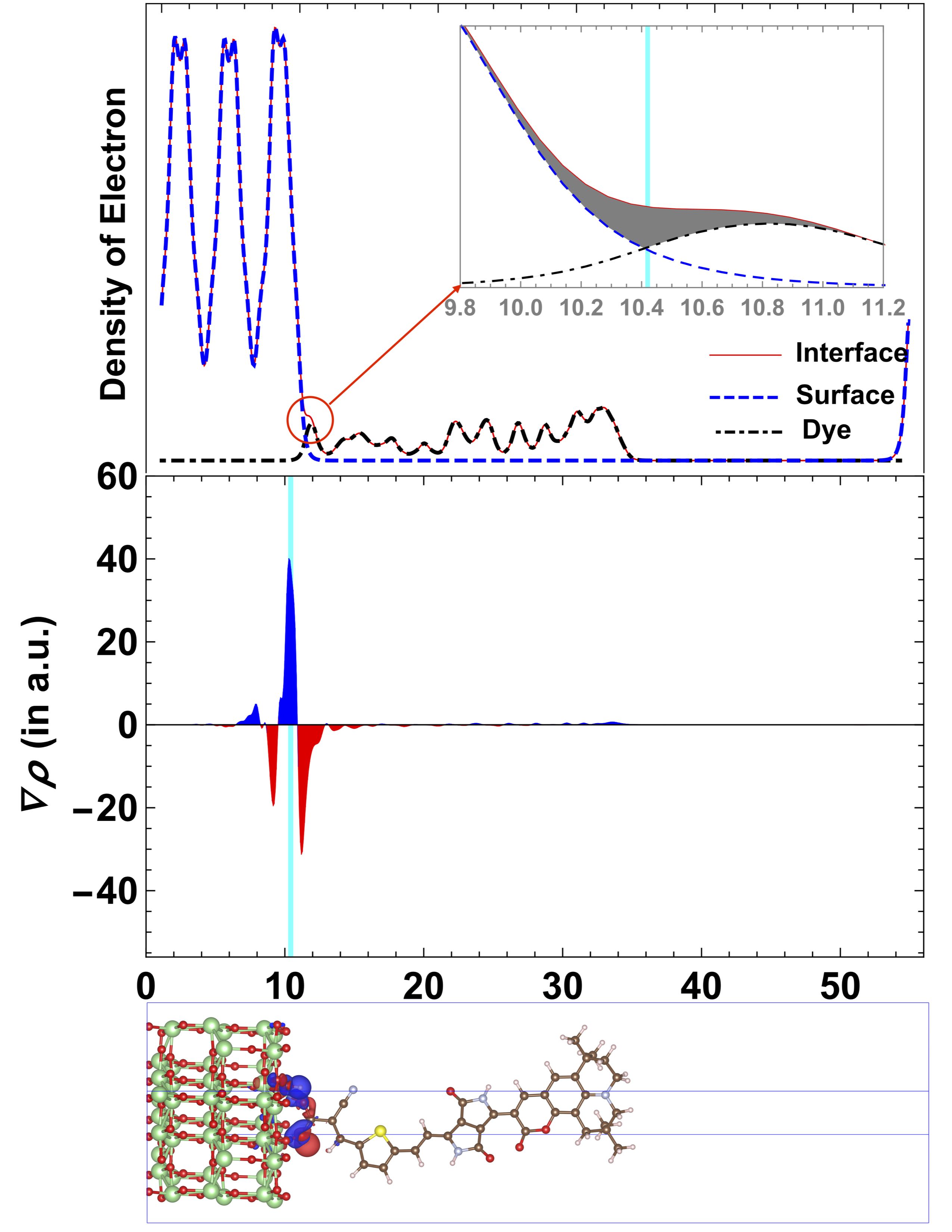


**Figure S4: For THQ5**


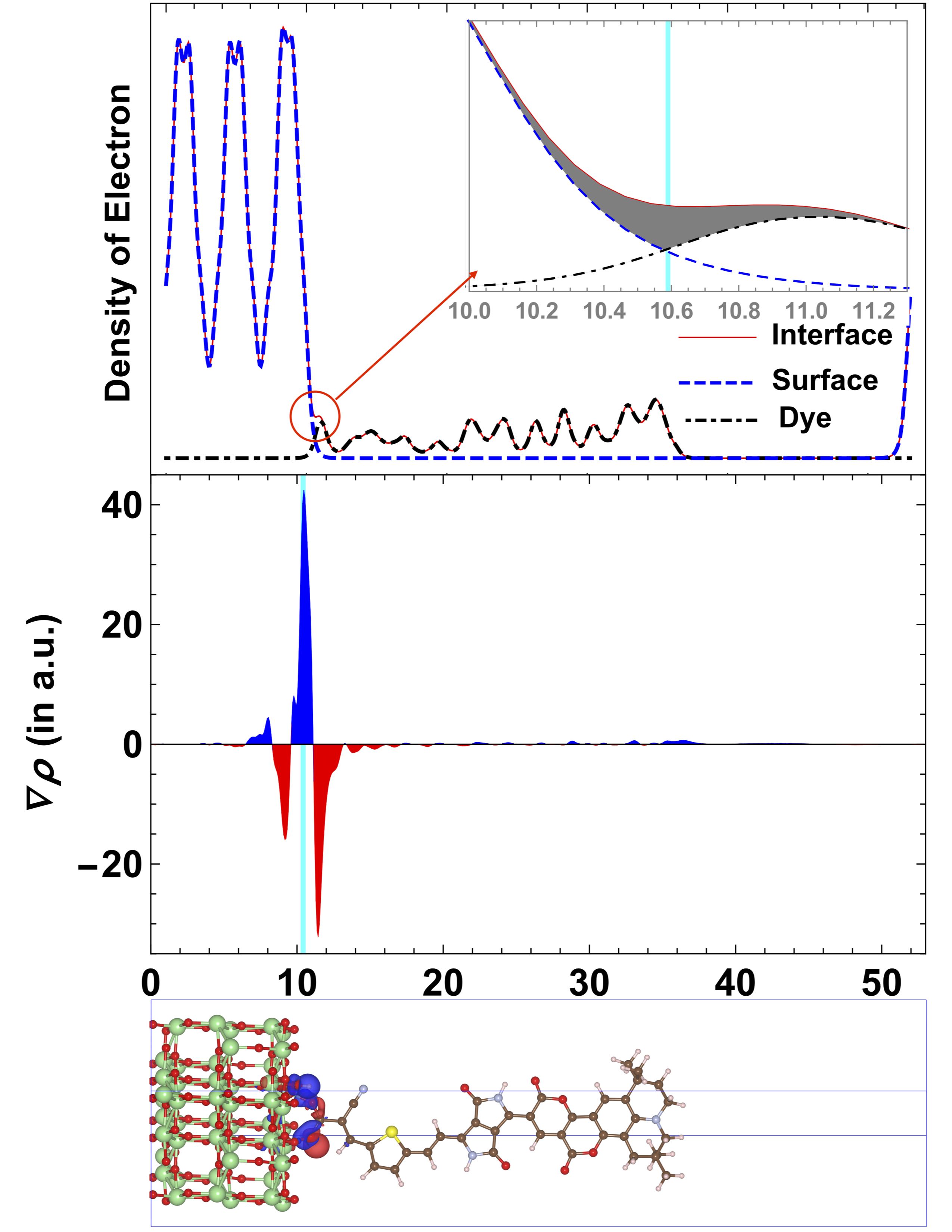


**Figure S5: For THQ7**


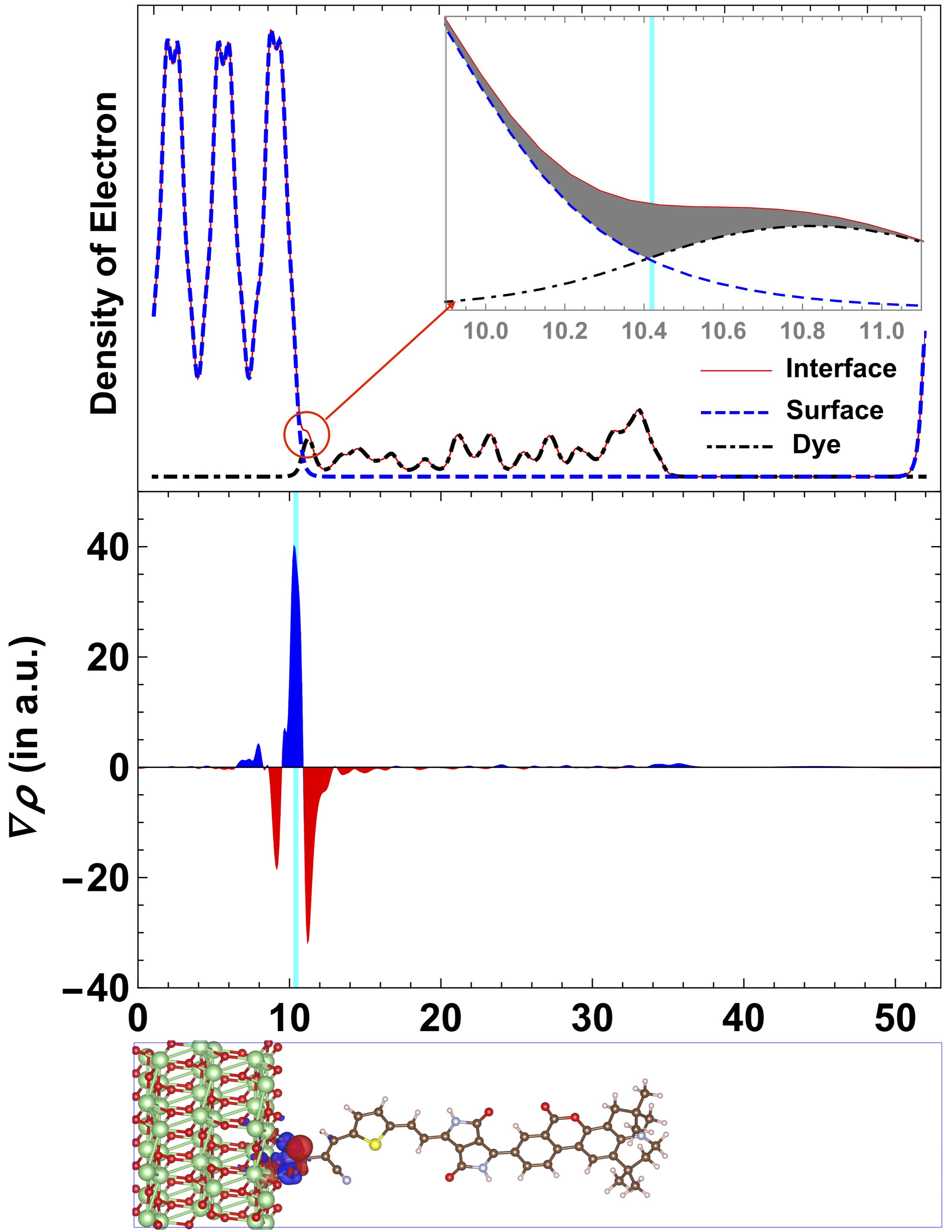


**Figure S6: For THQ8**


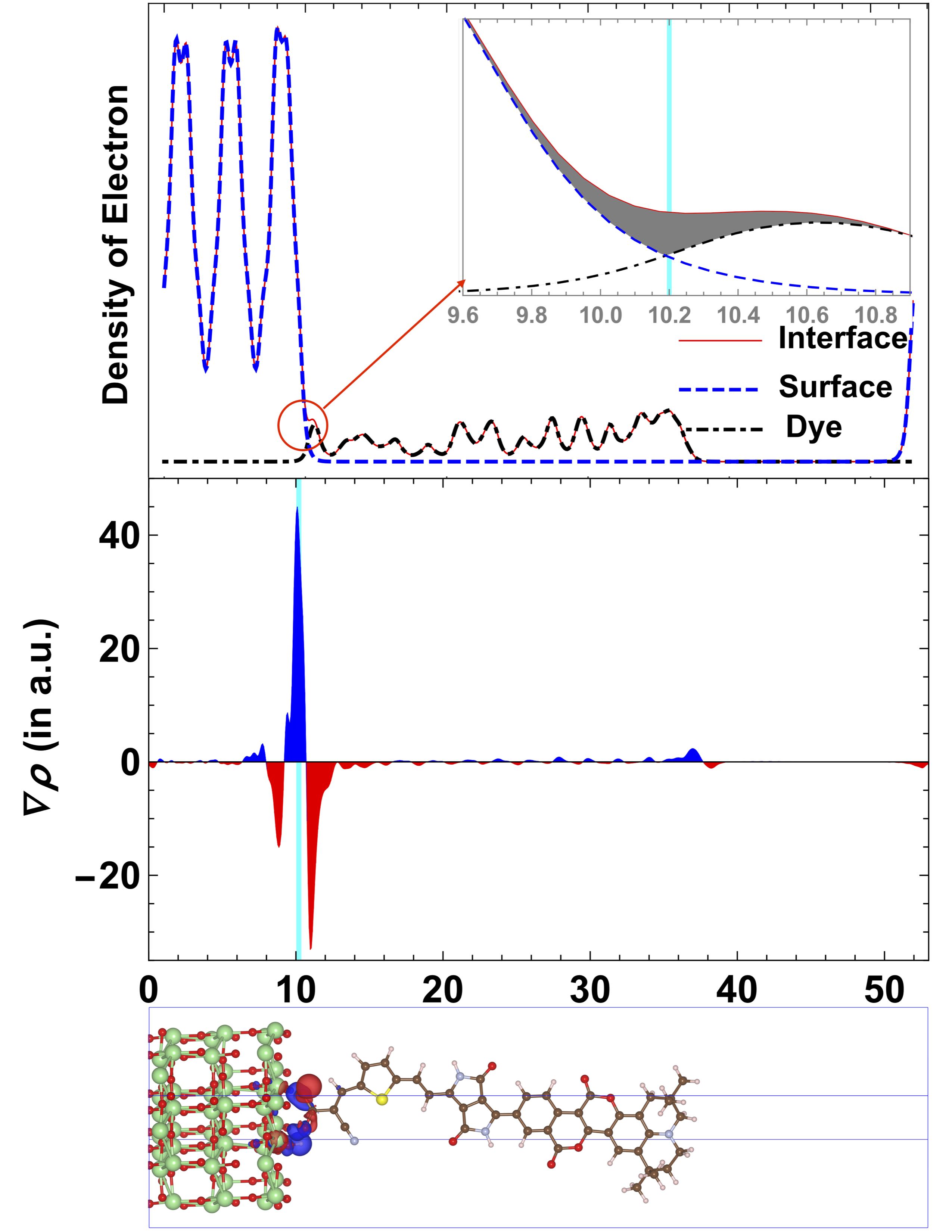


**Figure S7: For THQ9**


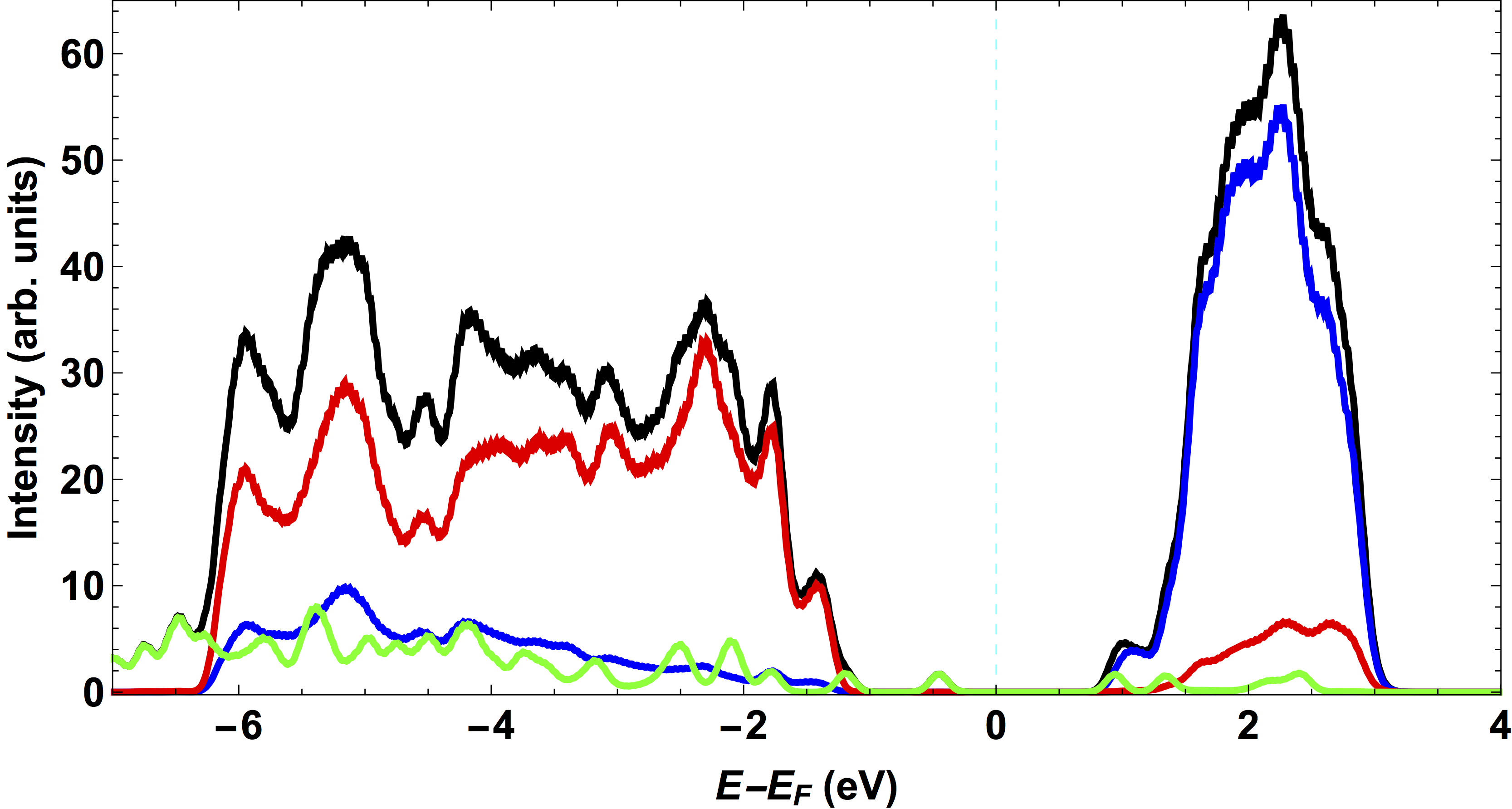


**Figure S8**: PDOS of the DI293. Black, blue, red & green representing the dye@TiO_2_, Ti-3d, O-2p and adsorbed dye, respectively.

**Table S1**: Bond lengths (in Å) of I-X (X= I, S, N) in dye---I_2_ complex of the studied isolated dyes.

| **Dye - - I_2_** | **CN - - I_A_** | **I_A_ - - I_B_** | **S_1_ - - I_C_** | **I_C_ - - I_D_** | **S_2_ – I_E_** | **I_E_ - - I_F_** |
| --- | --- | --- | --- | --- | --- | --- |
| THQ1 | 2.806 | 2.896 | 3.288 | 2.910 | 3.251 | 2.913 |
| THQ2 | 2.811 | 2.895 | 3.295 | 2.905 | 3.247 | 2.911 |
| THQ3 | 2.799 | 2.897 | 3.295 | 2.905 | - | - |
| THQ5 | 2.788 | 2.898 | 3.338 | 2.912 | - | - |
| THQ7 | 2.826 | 2.893 | 3.336 | 2.907 | - | - |
| THQ8 | 2.818 | 2.894 | 3.316 | 2.910 | - | - |
| THQ9 | 2.834 | 2.892 | 3.310 | 2.907 | - | - |

**Table S2:** Ab intio (VASP) calculated band gap, in eV, before (*E_g1_*) and after (*E_g2_*) adsorption off dye molecules with their difference (*ΔE_g_*).

| **Name** | ***E_g1_* (eV)** | ***E_g2_* (eV)** | ***ΔE_g_* (eV)** |
| --- | --- | --- | --- |
| THQ1 | 0.63 | 0.13 | 0.60 |
| THQ2 | 0.74 | 0.16 | 0.58 |
| THQ3 | 0.86 | 0.26 | 0.40 |
| THQ5 | 0.53 | 0.29 | 0.24 |
| THQ7 | 0.42 | 0.18 | 0.24 |
| THQ8 | 0.54 | 0.36 | 0.18 |
| THQ9 | 0.56 | 0.34 | 0.22 |
